# A Novel L-Shaped Fluorescent Probe for AIE Sensing of Zinc (II) Ion by a DR/NIR Response

**DOI:** 10.3390/molecules26237347

**Published:** 2021-12-03

**Authors:** Rosita Diana, Ugo Caruso, Francesco Silvio Gentile, Luigi Di Costanzo, Barbara Panunzi

**Affiliations:** 1 Department of Agriculture, University of Napoli Federico II, 80055 Portici, Italy; rosita.diana@unina.it (R.D.); luigi.dicostanzo4@unina.it (L.D.C.); 2 Department of Chemical Sciences, University of Napoli Federico II, 80126 Napoli, Italy; ugo.caruso@unina.it (U.C.); francesco.gentile@unina.it (F.S.G.)

**Keywords:** fluorescence, AIE, DR/NIR, zinc (II), sensing

## Abstract

In the field of optical sensors, small molecules responsive to metal cations are of current interest. Probes displaying aggregation-induced emission (AIE) can solve the problems due to the aggregation-caused quenching (ACQ) molecules, scarcely emissive as aggregates in aqueous media and in tissues. The addition of a metal cation to an AIE ligand dissolved in solution can cause a “turn-on” of the fluorescence emission. Half-cruciform-shaped molecules can be a winning strategy to build specific AIE probes. Herein, we report the synthesis and characterization of a novel L-shaped fluorophore containing a benzofuran core condensed with 3-hydroxy-2-naphthaldehyde crossed with a nitrobenzene moiety. The novel AIE probe produces a fast colorimetric and fluorescence response toward zinc (II) in both in neutral and basic conditions. Acting as a tridentate ligand, it produces a complex with enhanced and red-shifted emission in the DR/NIR spectral range. The AIE nature of both compounds was examined on the basis of X-ray crystallography and DFT analysis.

## 1. Introduction

Chemosensors involving an optical transduction within the visible spectrum (absorbance and/or fluorescence signal) are desirable naked-eye tools. Advanced bioimaging technology and optoelectronics have encouraged the quick advancement of fluorophores with deep red/near-infrared (DR/NIR) emission (from 650 to 900 nm), which could enhance the penetration and avoid the spectral auto-fluorescence overlapping in the living tissues [1,2,3]. However, the lower energy gaps of the red emitters usually lead to larger vibronic coupling between ground and excited states. This phenomenon increases the radiationless decay rate of the excitons and decreases the photoluminescence quantum yield (PLQY) of the probe, according to the energy gap law. Therefore, the design of efficient DR/NIR emitters is still a huge challenge [4,5].

Small molecules responsive to metal cations by a spectroscopic output are a specific and attractive research area [6]. Selective real-time response to metal ions attracts interests, due to their role in chemical and biological processes, and to their environmental impact [7,8]. In this context, organic fluorophores exhibiting fluorescence “turn-on” are highly sought-after tools. An emerging valuable building block for the design of responsive probes are small organic AIEgens. The luminescence response of AIE sensors at high concentrations or in the aggregated state can solve the problems due to the ACQ molecules, scarcely emissive as aggregates in aqueous media and in living tissues. The generalized mechanism explaining the emission of the AIEgens is named “RIM” (restriction of intramolecular motions) [9,10,11,12,13]. This effect includes rotation (RIR effect, restriction of intramolecular rotations) and other intramolecular motions concurring to the radiationless decay pathways. In presence of a metal cation, the AIE probe can undergo complexation equilibria, thus providing specific strategies in metal ion sensing [2,6,14,15,16,17,18,19,20,21]. Specifically, when an organic AIE probe is dissolved in solution, a turn-off of fluorescence is expected. The addition of a metal cation to the organic AIEgen dissolved in solution can cause a “turn-on” of the fluorescence by different mechanisms, involving the formation of emissive aggregates and implying the restriction of intramolecular motions and the metal-caused blocking of nonradiative pathways [9,17,22,23]. 

Thanks to a careful selection of the organic ligand, the specificity of the probe can be modulated as well as the response in the desired spectral zone. Cruciform- and half-cruciform-shaped probes are good candidates for metal capturing and recognization [22,23]. Molecular cruciform and half-cruciform cross-conjugated molecules have two conjugation circuits intersecting at a central core structure and can localize the highest occupied molecular orbital (HOMO) and the lowest unoccupied molecular orbital (LUMO) on different branches [22,24,25]. By the suitable assembly of electron donor (D) and electron acceptor (A) blocks connected through π-conjugated frameworks, the intramolecular charge transfer (ICT) process from D to A zone can be tuned. Therefore, the spectroscopic properties [24,26,27] involving intercrossed excited states between the low-lying local exciton (LE) and charge transfer (CT) exciton can be tailored and modulated. The sensing ability results from the metal binding, which affects the HOMO–LUMO gap and therefore the optical properties. 

The zinc (II) cation is well known for its ability to enhance a probe’s emission upon coordination (chelation enhancement effect, CHEF) [25]. As a closed-shell cation, it acts as a constraint, locking the ligand into an emissive conformation mainly due to LCT (ligand charge transfer) transitions, even altering intensity and/or position of the photoluminescence (PL) pattern [25,28,29,30,31,32,33,34]. With an AIE probe, zinc cation can recover and/or modulate the emission [25,35,36,37,38,39,40] by reformulating the orbital separation between HOMO and LUMO regions. Interestingly, zinc (II) has a potential in enhancing and red-shifting the emission of DR/NIR probes [1,2,3,41,42]. 

**Scheme 1:**
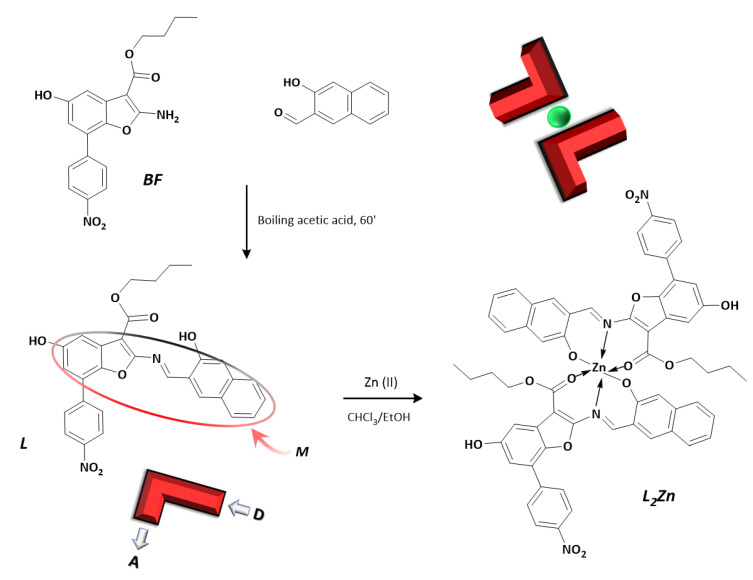
Synthetic route to the target compounds L and L_2_Zn. M is the main plane containing the benzofuran ring condensed with the 3-hydroxy-2-naphthaldehyde moiety. The cartoon representation is used for showing a quick glance of the donor–acceptor L-shaped pattern in the ligand L and in the complex L_2_Zn.

In the past years, our research group focused on the design and study of benzofuran- and benzodifuran-based architectures due to their optoelectronic, PL, and nonlinear optical properties, useful for biological and pharmacological applications [27]. We also employed the benzodifuran core to build cruciform organic fluorophore with a strong red emission due to RIR effect [43,44]. Herein, we report the synthesis and characterization of a novel benzofuran-based fluorophore obtained by condensation reaction between butyl 2-amino-5-hydroxy-7-(4-nitrophenyl)benzofuran-3-carboxylate and 3-hydroxy-2-naphthaldehyde. The molecule L in Scheme 1 has a half-cruciform or L-shaped pattern imposed by the conjugated main plane (M, marked in Scheme 1) and the crossed nitrobenzene moiety. The novel fluorophore proved to be an AIEgen with relevant DR emission in the solid state and in concentred solutions. Its half-salen functional group is a potential N,O-binding site for metal cations. In addition, the carboxylic oxygen group can participate in a tridentate coordination mode [38,45,46,47,48]. A fast colorimetric and fluorescence response was produced by adding zinc (II) salts to the organic ligand, both in neutral and basic conditions. The emission significantly increases and red-shifts as a consequence. 

In this work, the AIE nature of both L and its complex L_2_Zn were examined, and the mechanism is explored and discussed on the basis of X-ray crystallography and DFT calculations. The ligand L and its complex (L_2_Zn, see Scheme 1) were isolated and analyzed, and the AIE responsiveness of the ligand upon zinc binding was explored. In contrast to other metal cations expected in a biological context, zinc (II) cation was found to promote a selective “turn-on” of the fluorescence emission of the ligand dissolved in solution. The scarce interference due to other metals and the quantitative relationship make the ligand L a potential chemosensor for zinc (II) ion in biological environments. 

## 2. Materials and Methods

Commercially available starting products were purchased from Sigma Aldrich. Compound BF, butyl 2-amino-5-hydroxy-7-(4-nitrophenyl)benzofuran-3-carboxylate, was obtained as described in [43,49]. 

^1^H NMR spectra were recorded in DMSO-d_6_ with a Crystals 2020, 10, 269 4 of 15 Bruker Advance II 400MHz apparatus (Bruker Corporation, Billerica, MA, USA). Mass spectrometry measurements were performed using a Q-TOF premier instrument (Waters, Milford, MA, USA) with an electrospray ion source and a hybrid quadrupole-time of flight analyzer. Optical observations were performed by using a Zeiss Axioscop polarizing microscope (Carl Zeiss, Oberkochen, Germany) equipped with an FP90 Mettler microfurnace (Mettler-Toledo International INC MTD, Columbus, OH, USA). The decomposition temperatures (5 wt % weight loss), phase transition temperatures and enthalpies, and zinc content (determined as ZnO) were measured by employment of a DSC/TGA Perkin Elmer TGA 4000 (PerkinElmer, Inc., Waltham, MA, USA), scanning rate 10 °C/min. Absorption and UV-visible emission spectra were recorded by JASCO F-530 and FP-750 spectrometers (scan rate 200 nm min^−1^, JASCO Inc., Easton, MD, USA) and on a spectrofluorometer Jasco FP-750 (excitation wavelengths set at the absorption maxima of the samples, scan rate 125 nm min−1, JASCO Inc., Easton, MD, USA). Thin films of the neat samples were prepared using a SCS P6700 spin coater operating at 600 RPM for 1 min. Photoluminescence quantum efficiency values were recorded on quartz substrates by a Fluorolog 3 spectrofluorometer (Horiba Jobin Instruments SA), within an integrating sphere provided by an optical fiber connection. 

### 2.1. Synthesis of L

The synthesis of L was performed by dissolving 0.370 g (1.00 mmol) of BF at 70 °C in 15 mL of glacial acetic acid. A total of 0.344 g (2.00 mmol) of 3-hydroxy-2-naphthaldehyde was added under stirring. After 1 h at boiling temperature, the crude product precipitated. The compound was recovered from the hot solution and washed in methanol twice. Yield = 70%. Tm = 233 °C; Td = 255 °C. ^1^H NMR (400 MHz, DMSO-d_6_, 25 °C, ppm): 0.78 (t, 3H), 1.13 (m, 2H), 1.22 (m, 2H), 3.69 (t, 2H), 7.10 (d, 1H), 7.20 (d, 1H), 7.47 (t, 1H), 7.62 (d, 4H), 7.91 (d, 1H), 8.09 (d, 1H), 8.27 (d, 2H), 8.61 (d, 1H), 9.74 (s, 1H), 9.92 (s, 1H), 14.41 (s, 1H). Elemental analysis calculated (%) for C_30_H_24_N_2_O_7_: C, 68.70; H, 4.61; N, 5.34; found: C, 69.60; H, 4.90; N, 5.62. MALDI-TOF of L *m/z*: 525.3 (M + H).

### 2.2. Synthesis of L_2_Zn

The synthesis of L_2_Zn complex was performed by dissolving 525 mg (1.00 mmol) of L in 20 mL of hot CHCl_3_, and 68.1 mg (0.500 mmol) of ZnCl_2_ (dissolved in ethanol) was added drop by drop. The solution was kept under reflux for 1 h. When the solution was cooled, a dark red crystalline solid was obtained. The compound was recovered by filtration and washed twice in cold methanol. Yield = 88%. Td = 320 °C (decomposition of the solid sample). ^1^H NMR (400 MHz, DMSO-d_6_, 25 °C, ppm): 0.85 (t, 3H), 1.20 (m, 2H), 1.31 (m, 2H), 3.81 (t, 2H), 7.09 (d, 1H), 7.18 (d, 1H), 7.46 (t, 1H), 7.52 (d, 1H*)*, 7.62 (m, 3H), 7.79 (d, 1H), 7.91 (d, 1H), 8.27 (d, 1H), 8.36 (d, 2H), 9.64 (s, 1H), 9.80 (s, 1H). Elemental analysis calculated (%) for C61H49N4O14Zn: C, 64.98; H, 4.38; N, 4.97; found: C, 65.23; H, 4.60; N, 5.26. MALDI-TOF of L_2_Zn *m*/*z*: 1126.3 (M + H).

### 2.3. Job’s Plot Measurements

Job’s plot measurement of L_2_Zn was performed on 500 μM solutions of zinc (II) chloride in water and 500 μM of L in ethanol. Volumes of 3.00, 2.75, 2.50, 2.00, 1.50, 1.00, 0.50, 0.25, and 0 mL of the ligand solution were used and added to 0, 0.25, 0.50, 1.00, 1.50, 2.00, 2.50, 2.75, and 3.00 mL of the metal, with each vial having 3.0 mL total volume.

### 2.4. UV-Visible Titrations of L with Zinc (II) Cation and Naked-Eye Analysis for the Adduct L_2_Zn

A stock solution (0.1 M) of chloride salt zinc (II) ion in ethanol was prepared. Solutions of L (500 μM in ethanol) were prepared. Titrations were performed by adding 0.5 μL of stock solution of zinc salt to 2 mL of L solution. For the naked-eye analysis, 10 samples were prepared by adding zinc salt in growing increments. Specifically, Zn:L ratio grew from 0.05:1 to 0.5:1 in the vials used for the photos.

### 2.5. Single-Crystal X-ray Analysis

Crystals of L were obtained at room temperature by slow evaporation from an ethanol solution (about 500 μM, 2 mL). Red crystals of L appeared as small plates with typical dimensions of 0.03 × 0.04 × 0.3 mm. Single dark red crystals of form L_2_Zn were obtained at room temperature by slow evaporation of stoichiometric mixture of L in ethanol (500 μM, 2 mL) and zinc acetate (II) in water (20 mM, 0.050 mL), at pH < 7. Data of L and L_2_Zn were collected with synchrotron radiation (wavelength, 0.7000 Å) from XRD1 beamline at the Elettra Synchrotron Light Source, Trieste Italy. 

By using a small loop of fine rayon fiber, we dipped selected crystals for data diffraction, of both compounds, in cryoprotectant Paratone oil and flash-frozen in a stream of nitrogen at 100 K. Despite very small dimensions of L molecule crystals, a complete data set was recorded. Data were processed using XDS and POINTLESS 1.11.21, with data collection statistic reported in Section 3.4 [50]. No data twinning was detected. Crystals of L had a triclinic unit cell with axes a = 6.13 Å, b = 14.99 Å, c = 16.24 Å and space group *P* 1, while L_2_Zn showed a monoclinic unit cell with axes a = 15.99 Å, b = 15.20 Å, c = 22.15 Å. Structures of L and L_2_Zn were found by direct methods using SHELXS [51], which revealed the expected skeletons corresponding to two distinct molecules in the ASU. Structures were anisotropically refined using full matrix least-squares methods on F^2^ against all independent measured reflections using SHELXL [52] run under WinGX suite for the refinement of small molecules [53]. SIMU and/or ISOR as implanted in SHELXL were used for the refinement of L molecule. Hydrogen atoms were introduced and refined in agreement with a riding model as implemented in SHELXL. Crystal data and structure refinement details for L and L_2_Zn complex crystallized and a more exhaustive discussion are reported in Section 3.4 and in the table contained therein. Figures were generated using Mercury CSD 3.6 [54] Crystallographic data of L and L_2_Zn complex have been deposited with the Cambridge Crystallographic Data Centre and can be obtained via www.ccdc.cam.ac.uk/data_request/cif, (accessed on 26 November 2021).

### 2.6. DFT Computational Details

Ab initio periodic 3-D models, derived from X-ray crystallographic data, were developed for L and L_2_Zn system. The software package utilized is a development version of *Crystal17* code [55], where density functional theory (DFT) methods are implemented, adopting linear combination of atomic orbital (LCAO) formalism. In LCAO approximation, each crystalline orbital is a linear combination of Bloch functions, defined in terms of local atomic orbitals. A hybrid functional B3LYP [56,57] with Grimme D3 empirical correction [58] was adopted to obtain an improved evaluation of the exchange and dispersion interactions. The basis set utilized for H, C, O, N, and Zn atom groups are the pob_TZVP_rev2 [59]. This basis set, derived from Ahlrichs basis set [60], can be considered very reliable for the evaluation of electronic properties of this family of molecular crystals.

The space group of L and L_2_Zn systems are *P* 2_1_ (to simplify the theoretical implant, we used P1) and *P* 2_1_/n, with 2 and 4 symmetry operators, respectively. The number of atoms and of atomic orbital in the asymmetric unit cell are, respectively, 142 and 1964 (for L) and 500 and 6880 (for L_2_Zn). The symmetric irreducible part of the Brillouin zones was sampled using a Γ-point-centered Pack-Monkhorst grid of 13 and 8 k-points for L and L_2_Zn, respectively. The electron density was integrated not by using the default pruned grid with 75974 points but adopting the most performing grid with 991454 points (99 radial and 1454 angular points). Other technical details (SCF energy convergence, Coulomb and Exchange truncation series and Gradient tolerance threshold) were described in detail in our previous paper [61]. Some electron properties, such as band gap and electron density isosurface of the crystallin orbitals (COs), were calculated from the wavefunction of the optimized geometries. 

## 3. Results and Discussion

### 3.1. Design and Spectroscopic Behaviour of L

The synthetic route for the benzofuran precursor BF in Scheme 1 followed a reported procedure [49] consisting in the diazotization of 4-nitroaniline and the coupling of the diazonium salt on a benzoquinone. The target compound L is the condensation product between the aminic functional group of BF and the bulky salicylic naphthaldehyde. By choosing a medium-length carboxylic chain on the benzofuran core, we guaranteed solubility, improved the RIR effect, and produced the third binding site. The donor naphthol core fused to BF enlarges the conjugated length and enriches the aromatic pattern [43]. The intramolecular H-bond interactions between the iminic nitrogen atom group and the oxygen atom group of the phenol in the naphthol moiety further stiffened the main plane (M in Scheme 1), with which the nitrophenyl ring was twisted (Section 3.4). The compound L has attracted attention as an AIEgen, as a DR/NIR fluorophore, and as a nitro-substituted aromatic emitter. In fact, the nitro group is widely recognized as a strong fluorescence quencher, usually lowering LUMO energy and causing the intramolecular photoinduced electron transfer (PET) process from the excited fluorophore to the electron-withdrawing nitro-aryl moiety. In our case, the nitro group narrows the spatial separation of frontier molecular orbitals of the two regions (the electron-donor main plane and the electron-acceptor nitrophenyl moiety), not so much to quench the emission but moving the PL band to the DR/NIR spectral zone. As a result, L is a red dye and a deep red-emissive fluorophore.

**Figure 1:**
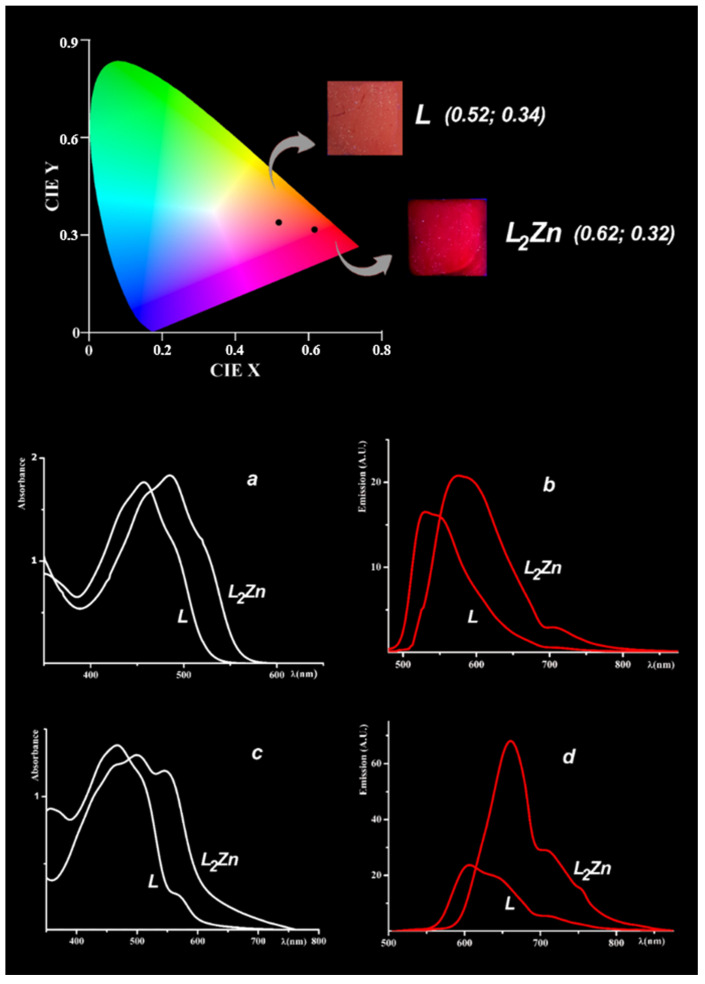
Above: emission CIE diagram of L and L_2_Zn in the solid phase. The samples used to record emission spectra and to calculate emission CIE coordinates are pictured in the insets. Below: absorption spectra (white curves) and emission spectra (red curves) of L and L_2_Zn in 0.1 mM TCE solution (**a**,**b**) and in the solid phase (**c**,**d**).

The compound L underwent a spectroscopic analysis by absorption and UV-visible emission spectrophotometry in solutions and on thin films of finely crumbled crystals spin-coated onto quartz slides (results summarised in Table 1). In TCE solution, the absorbance pattern revealed a broad band peaked at 457 nm, red-shifted at 468 nm for the crystalline sample. The emission spectrum of the sample in TCE (see Figure 1) irradiated at the absorbance maximum showed a humped peaked band at 533 nm with 76 nm Stokes shift. TCE was elected for most experiments to compare ligand and complex. In other common organic solvents where L is highly soluble (as chloroform, acetone, ethyl acetate, DMSO), the PL pattern is largely preserved. As expected, PLQY measured in diluted TCE solution was scarce (less than 1.5%). In the solid state, the emission of a crystalline L sample (see Figure 1) showed a large part of the band in the DR region with a maximum at 608 nm and an appreciable part in the NIR region (above 700 nm). A large Stokes shift was detected (139 nm, see Table 1, measured from the absorbance maximum to the emission maximum), which involved a scarce reabsorption of the emitted photons and therefore high emission efficiency. PLQY recorded on the same crystalline thin layer was 7.5%, to be considered a good result for a DR/NIR emitter [19]. 

**Figure 2:**
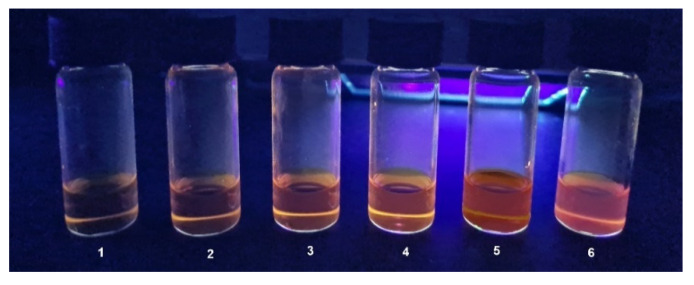
The AIE self-assembly naked-eye test as can be appreciated under an UV-visible lamp at 365 nm. Vial 1: 0.1 mM TCE solution of compound L. Vials 2-6 TCE/hexane 0.1 mM solutions in the increasing ratio: 20, 40, 60, 70, 80%.

As expected for an AIEgen, the aggregation process involving the self-assembly of the emissive units improved fluorescence emission so that it could be modulated by a different solvent/non-solvent ratio. In our case, the easy and fast naked-eye test [62,63,64] was performed in TCE/hexane-diluted solutions (0.3 mM). In TCE-diluted solution, the sample was scarcely emissive. As the percentage of hexane increased, a red fluorescence was gradually perceptible in the solution, due to the formation of emissive aggregates. The effect can be appreciated under the UV-visible lamp at 365 nm and is shown in Figure 2. The fluorescence gradually increased up to 60% (vial 4) and became evident in the 70% hexane solution (vial 5). After 70% (80% hexane solution in vial 6), some extent of a precipitated solid was observed at the bottom of the vial, although the suspended part still had an obvious red fluorescence.

### 3.2. Synthesis and Spectroscopic Behaviour of L_2_Zn

The probe L contains a half-salen [12,57,58,59] site potentially working as a N,O chelating site. Upon coordination to a metal cation, the probe is expected to undergo conformational and electronic changes, leading to specific remodeling of the absorbance and emission bands. The Schiff-base pattern has been demonstrated to be a winning strategy in building AIE coordination complexes [65,66,67,68,69] with suitable metal cations. The presence of the carboxylic oxygen further supports the coordination pattern due to the formation of a tridentate pincer [20,38,70,71,72]. By addition of both neutral (chloride) or basic (acetate) zinc (II) salts, the formation of the complex L_2_Zn was observed in real time. For this study, we isolated the complex L_2_Zn by reaction of the ligand L with zinc acetate in TCE/ethanol. Deep red crystals were easily obtained by concentering and cooling the solution. As is discussed in Section 3.4, the asymmetric unit of L_2_Zn contained a complex of Zn (II) with two ligands acting as mononegative tridentate chelators. In the complex, the main plane M underwent a slight bending to arrange the octahedral geometry around Zn^2+^. Zinc cation acted as a constraint, blocking the coordinated ligand in a structural pattern involving a different HOMO–LUMO bandgap and intercrossed excited states (see Section 3.5). 

Absorbance and emission spectra of the complex (see Table 1) showed a red-shift of the maximum both in solution and in the solid phase. A diluted TCE solution of L_2_Zn showed the absorbance maximum at 477 nm and the emission maximum at 578 nm, with Stokes shift at about 100 nm. Like the unbonded ligand, PLQY measured in diluted TCE solution was scarce (about 1/3 of the band is above 700 nm). In concentrated solution and in the solid state, the complex was a DR pigment and a DR/NIR emissive fluorophore. The absorbance of a crystalline sample of L_2_Zn deposed onto a quartz slide peaked at 500 nm. The emission of the same sample showed a broad band in the DR region with a maximum at 661 nm; a relevant part of the emission spectrum fell in the NIR region (above 700 nm). The Stokes shift related to the intramolecular relaxation process from LE state to the ICT was large (about 160 nm) [70] and led to a distinct DR/NIR emission (see CIE coordinates in Table 1 and in Figure 1). Interestingly, the complex was proven to be a novel, more efficient DR/NIR AIEgen with respect to L ligand. PLQY measured on a crystalline thin layer of L_2_Zn was 22%, a remarkable result for a DR/NIR emitter. 

The red-shift and the fluorescence enhancement in the spectral pattern of the complex with respect to the unbonded ligand was not unexpected. A study on both phenomena was inspired by the red-shift observed in yellow fluorescent proteins due to the formation of π−π stacking, as well as the enhanced fluorescence intensity observed in green fluorescent proteins due to the structural stiffening imposed by zinc (II) cation [73]. Several D–A type asymmetrical salen ligands were found to undergo a red-shift and fluorescence enhancement upon zinc (II) coordination [74]. In fact, the stronger interactions in the organic part promoted by the coordination can induce the overlap of electron clouds in the electron-rich moieties, with the extension in conjugation, promoting a red-shifted spectra [75]. On the other hand, the fluorescence enhancement can be ascribed to the closed-shell zinc cation that locks the organic ligand and stabilizes the excited state by carrying out the CHEF mechanism [27].

As shown in Section 3.5, the spectroscopical pattern was analyzed on the basis of the DFT calculations. From the calculated pattern, some considerations regarding the optical behavior were made, starting from the narrow HOMO–LUMO band gap and the confirmed the “clip” role of the zinc cation. 

### 3.3. Responsiveness of the AIE Probe L to Zinc (II) Cation

Due to its essential biological role, many AIE-based fluorescence sensors for Zn^2+^ ion have been developed and applied [14,18,76,77,78,79,80,81,82,83,84,85,86,87]. A metal cation can induce a fluorescence turn-on of the AIE probe dissolved in solution by different mechanisms: primarily, the formation of emissive aggregates (cleavage-triggered aggregation, CTA). In addition, we must consider the restriction of intramolecular motions (chelation-aided rigidification, CAR), the metal-caused blocking of nonradiative pathways (metal-bridged crosslinking, MBC), and the metal-caused blocking of intramolecular motion (coordination-induced complexation; CIC) [17,20,88,89,90,91,92,93,94,95]. As zinc (II) ion acts as a spectroscopically silent “clip” locking the ligands into an emissive conformation, we expected that the PL response of the L probe toward Zn^2+^ could be unique. 

As a preliminary test, we evaluated the fluorescence response of L to a group of metals of biological interest, including the most common and transition metals. The experiments were performed in ethanol as a water-miscible solvent, used to dissolve both the ligand and the inorganic salt (chloride). Absorbance and emission response of common alkali and alkaline earth metals (Na^+^, K^+^, Mg^2+^, Ca^2+^, Ba^2+^) and first-row transition metals (Cr^3+^, Mn^2+^, Fe^3+^, Co^2+^, Ni^2+^, Cu^2+^) in the presence of the ligand were recorded. By reaction with L, the non-transition metal cations produced no variation in the original absorption and emission spectra of the unbound ligand. All the transition cations (Cr^3+^, Mn^2+^, Fe^3+^, Co^2+^, Ni^2+^, Cu^2+^, Zn^2+^) caused a red-shift of the absorbance maximum from 20 to 30 nm of 1.00 mM ethanolic solutions of L (about 50 nm in the case of Fe^3+^). On the other hand, the emission response of L upon coordination of Cr^3+^, Mn^2+^, Fe^3+^, Co^2+^, Ni^2+^, and Cu^2+^ recorded onto 1.00 mM ethanolic solutions pointed out a complete fluorescence quenching. Only in the case of zinc (II) ion was both a spectroscopical red-shift and an obvious fluorescence enhancement detected. 

On the basis of these preliminary data, we examined the fluorescence sensing performance of L involving the emission response of a 500 μM ethanol solution of L to ZnCl_2_ dissolved in 0.100 M ethanol solution. The salt was incrementally added, starting from 0.1:1 (Zn:L) up to 0.5:1 (stoichiometric amount). Data were collected to further confirm the expected stoichiometry in solution. Job’s experiment [96] was performed for the adduct L–Zn, and the plot is reported in Figure S1. As the maximum emission intensity underwent a red-shift, Job’s plot for the binding of L with zinc (II) was obtained by plotting emission intensity (at 600 nm) vs. mole fraction of metal ion and suggests a 1:0.5 (L:Zn) stoichiometry ratio for the complex ascribed.

**Figure 3:**
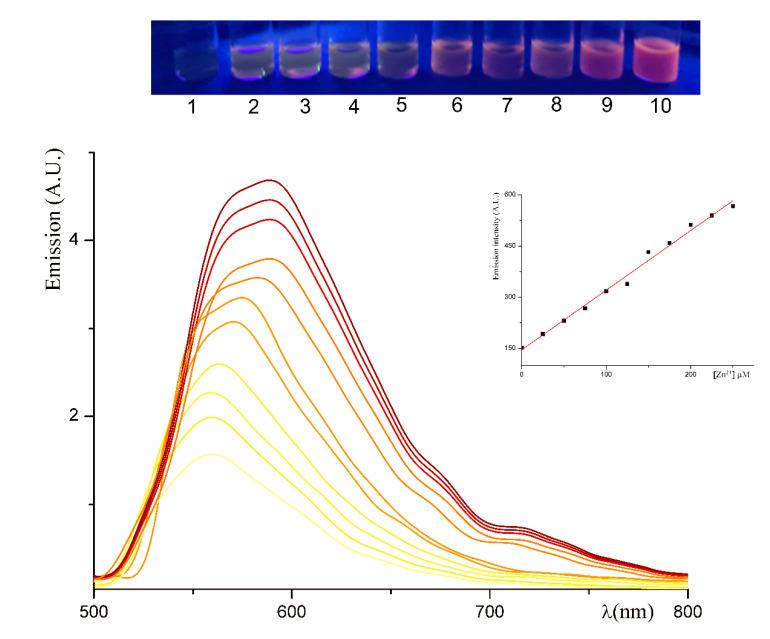
Fluorescence titration obtained by recording the emission spectra of 500 μM ethanolic solution of L and upon incremental addition of ZnCl_2_ (0.100 M in ethanol), excited at 450 nm. Inset above: naked eye-perceivable emission under a UV–VIS lamp operating at 365 nm of the samples obtained by growing increments of the Zn:L ratio. Specifically, the vial marked zero is the solution of the unbonded ligand; in the vials, 1–10 Zn:L ratio grew from 0.05:1 to 0.5:1. Inset below: the graphic of the relationship between emission intensity and concentration of Zn^2+^.

The unbounded ligand displayed a poor red emission (as can be observed in Figure 3). The incremental addition of the zinc salt dissolved in ethanol produced a fluorescence recovery due to the formation of emissive aggregates [16,17,40,97,98,99,100,101]. A gradual red-shift in the emission maximum was also recorded. The sensing mechanism can be ascribed to the CTA effect caused by nanoscale aggregates that increase in size up to the observation of a turbidity not involving precipitation (detectable under a 100× enlargement microscope starting from the sample 3 of Figure 3 and naked eye-perceivable starting from the sample 4 in Figure 3). A similar naked eye-perceivable pattern could be observed, starting from 100 μM solutions of L. Moreover, L molecule was found to be responsive in the AIE mode to both neutral (chloride) and basic (acetate) zinc salt, with an analogous emissive pattern. A roughly linear relationship (with correlation index = 0.91) between PL increase (evaluated as the area under the emission peak) and zinc concentration was found, suggesting the possibility of a quantitative use. 

The fluorescent emission spectra of L in the presence of the different cations together with Zn^2+^ were recorded to evaluate the interference to the zinc (II) binding. The addition of the unresponsive metal ions to a solution of L_2_Zn did not disturb the color/emission perception. Contrarily, an interference was recorded when solutions of L_2_Zn were mixed with the responsive transition metals. Specifically, the emission curve of L_2_Zn adduct (200 mM in ethanol) underwent a decrease from 15% to 30% in the presence of a stoichiometric amount of Cu^2+^, Co^2+^, Ni^2+^, Cr^3+^, and Mn^2+^. Only in the presence of the stoichiometric amount of iron did the emission band undergo the 80% decrease. The fluorescence spectra of L_2_Zn adduct in the presence of 1 equivalent of other interferent metal cations are reported in Figure S1 with the related interferogram. 

### 3.4. X-ray Structural Characterization of L and L_2_Zn Complex

**Figure 4:**
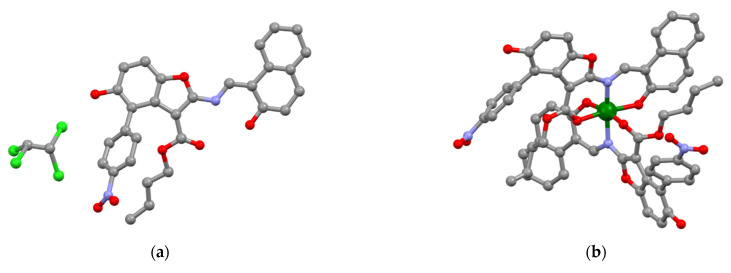
Ball-and-sticks representation of L (**a**) and L_2_Zn complex (**b**) crystal structures. Atoms are color-coded as follows: carbon (gray), oxygen (red), nitrogen (blue), zinc (dark green), chlorine (light green).

Crystals of L and L_2_Zn were obtained by slow evaporation at room temperature (see Section 2.5). The asymmetric unit of L contained one molecule and one 1,1,2,2-tetrachloroethane, as shown in Figure 4a. Some structural data are reported in Table 2. The L molecule was characterized by a main planar structure with the naphthol and benzofuran groups laying on a plane with an average displacement of the atoms of ≈0.03 Å. The iminic nitrogen atom group, the benzofuran hydroxyl group, and an oxygen atom group of the ester were oriented in a position for a possible coordination (Figure 4a). The L molecule showed an intramolecular N–H–O hydrogen bond in the half-salen group (O distance = 2.57 Å), enforcing the planarity of the system. The nitro-phenyl group was twisted of ≈45° with respect to the main plane of the benzofuran group to minimize interactions with the close bulky ethyl-ester group, slightly out from the main plane. This pattern was already observed in BDF molecules [43]. The hindered rotations satisfied the requirements of RIR molecules. The molecules of L presented a crystallographic head-to-tail antiparallel arrangement, and this orientation stabilized an intermolecular hydrogen bond (2.69 Å) of the benzodifuran hydroxyl group. Finally, several intermolecular Van der Waals interactions involving the aliphatic groups of the n-butyl chains and several atoms of the benzodifuran group and with the aromatic groups were observed.

**Figure 5:**
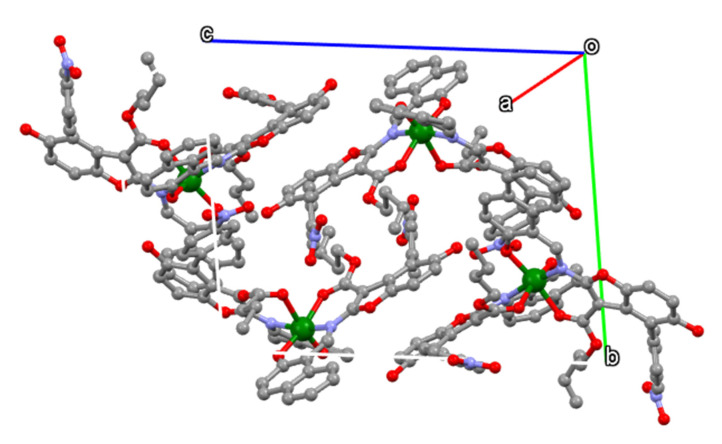
Crystal packing of L_2_Zn complex along **a** axis direction.

Dark-red crystals of L_2_Zn complex were obtained by slow evaporation at room temperature (see Section 2.5). The asymmetric unit contained one electroneutral dimeric complex of ZnL_2_ complex, as shown in Figure 4a. The dimer L_2_Zn showed an overall head-to-tail arrangement of L molecules in coordinating the zinc ion. The molecular structure of L_2_Zn displayed a distorted octahedral coordination to zinc ion. Specifically, zinc coordination sphere included the –N and –O atom groups of the half-salen of each L molecule, and its coordination sphere was completed by an oxygen atom group of the carbonyl ester. Notably, the presence of the butyl chain enforced one of the coordinating L molecules’ benzofuran group a rotation with respect to the average molecular plan. This rotation resulted in a higher coordination bond distance (2.27 Å) of O…Zn distance (average distance of 2.08 Å). Therefore, the coordination to the metal slightly changed the conformation when L ligand was compared to its metal-bound form. The crystal packing of L_2_Zn complex was stabilized by intermolecular Van der Waals interactions and strong π–π interactions of benzofuran group (average interplanar distance of ≈3.3 Å). A crystal packing arrangement of L_2_Zn molecules can be observed in Figure 5. 

### 3.5. DFT Analysis of L and L_2_Zn

The first relevant fact emerging from the DFT analysis was that both L and L_2_Zn systems exhibited a direct transition localized in Γ. Other relevant and expected data were the HOMO–LUMO band gap values, 2.60 eV and 2.46 eV for L and L_2_Zn compounds, respectively. The red-shift observed both in the absorption and emission pattern from the unbonded ligand L to the complex L_2_Zn can be ascribed to the 0.14 eV band gap reduction.

**Figure 6:**
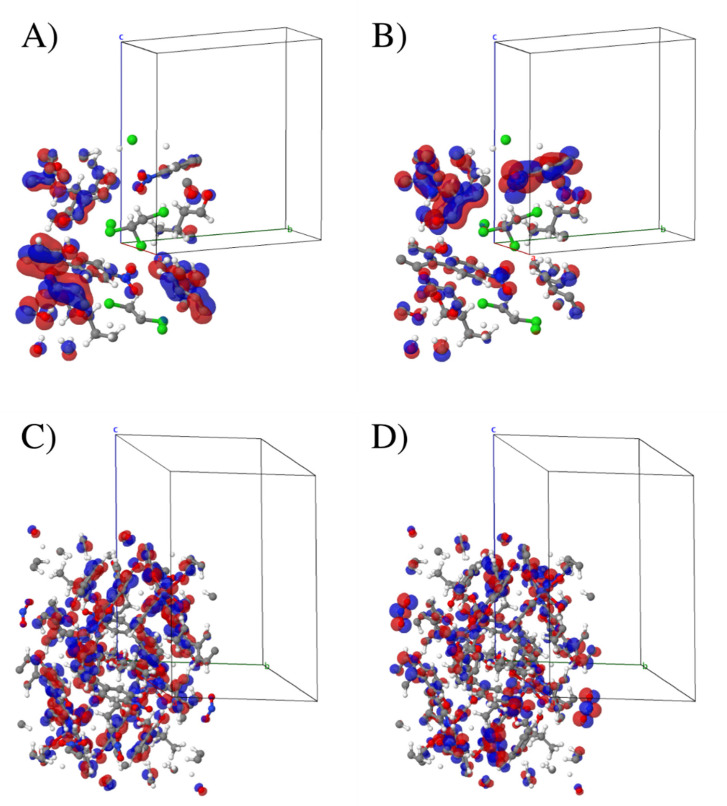
Orbital isodensity plot of the L system for HOCO (**A**) and LUCO (**B**), and of L_2_Zn for HOCO (**C**) and LUCO (**D**). The isolevel was set to 0.01 |e|/Bohr. The asymmetric unit cells had 142 and 500 atoms for the L and L_2_Zn compounds, respectively.

To evaluate the local character of the single molecular orbitals, we performed a band component analysis for the HOCO (highest occupied crystalline orbital) and LUCO (lowest unoccupied crystalline orbital) bands in both structures. From the band analysis in Γ, the frontier orbitals in both systems showed a delocalized behavior. A tolerance threshold of 0.01 was set to discriminate the most important eigenvector coefficient. The square modulus of each coefficient was compared with this tolerance, and terms with lower value were omitted (see ANBD directive in *Crystal17 manual*) [55]. Due to the system complexity, such analysis assigning a specific weight in terms of atomic orbital to each band was suitable for deriving the representation of Figure 6, in which two very different situations can be observed.

In L molecule, the main contribution of the HOCO band can be attributed to p_x_, p_y_, and p_z_ atomic orbital belonging to a restricted number of atoms in a specific region of the molecule. The mainly involved atoms belong to the benzofuran condensed rings (Figure 6A). In this band, the value of square modulus of each coefficient was in a range of 0.11 < |c| < 0.13. For L_2_Zn complex, the HOCO band involved many different sites in the asymmetric unit cell, as illustrated in Figure 6C. This band was much more delocalized and spanned in a large region of the organic ligand with the max value of |c| of 0.076, lower than the unbonded ligand L. In the L molecule, the main components were able to be attributed to the p_y_ atomic orbital of C, N, and O atom groups with 0.05 < |c| < 0.08. The LUCO band analysis of the L molecule showed an atomic orbital behavior localized on C and O atom groups. Specifically, the carbon atoms involved were different in the HOCO and in the LUCO. In addition, the p_y_ and p_z_ orbitals of the N atom groups were involved (Figure 6B). In the L_2_Zn system, the LUCO was mainly composed of p_y_ orbital of N atom groups (Figure 6D). These results confirmed the scarce role of zinc (II) cation in the frontier band and its negligible contribution to the atomic orbital. On the other hand, the metal preserved the fluorescence channel in the AIE ligand through a more delocalized pattern of the LCT bands.

## 4. Conclusions

The addition of zinc (II) cation to a half-cruciform benzofuran-based AIE ligand caused the increase and red-shift of the emission response. Acting as a tridentate ligand in a 2:1 (L:Zn) stoichiometric ratio, the novel organic DR/NIR ligand produced a DR/NIR AIE complex with about one-third of the emission band in NIR region and a respectable PLQY. The novel AIEgens L and L_2_Zn complex were fully characterized spectroscopically and studied by X-ray crystallography. DFT analysis was employed for a complete understanding of the spectroscopical behavior. The role of the zinc (II) cation in the activation of the AIE channel involved a more delocalized LCT pattern with respect to the unbound ligand. In contrast to other metal cations expected in biological environments, zinc cation did cause a gradual “turn-on” of the fluorescence emission of the AIE ligand dissolved in solution, due to the CTA effect. In conclusion, the selective fluorescence response, the scarce interference due to other bio-relevant metal cations, and the quantitative relationship between fluorescence increase and metal concentration in neutral and basic conditions made the probe a potential chemosensor for zinc (II) ion in a biological context.

## Figures and Tables

**Table 1:** Absorption and emission properties of L and L_2_Zn in solutions and in the solid state.

Sample	λ_ab_^(a)^	ε·10^3^	λ_em_^(b)^	λ_ab_^(^^c)^	λ_em_^(d)^	PLQY% ^(e)^	CIE ^(f)^
L	457	32.1	533	468	607	7.5	0.52; 0.34
L_2_Zn	477	32.9	578	500	661	22	0.62; 0.32

^(a)^ Wavelength of UV-visible absorbance maxima (nm), and related molar extinction coefficients, (M^−1^cm^−1^), in TCE. ^(b)^ Wavelength of emission maxima (nm) in TCE. ^(c)^ Wavelength of UV-visible absorbance maxima (nm) of the solid samples. ^(d)^ Wavelength of emission maxima (nm) of the solid samples. ^(e)^ PLQYs of the solid samples. ^(f)^ CIE measured in emission on the solid samples (as a fluorophore).

**Table 2:** Data collection and refinement statistics for L and L_2_–Zn complex.

	L	L_2_Zn
CCDC number	2123894	2120416
Asymmetric unit content	L⋅C_2_H_2_Cl_4_	L_2_Zn
Color	Red	Dark-red
Temperature (K)	100	100
Wavelength (Å)	0.7000	0.7000
Crystal system	Monoclinic	Monoclinic
Space group	*P* 2_1_	*P* 2_1_/n
*a* (Å)	6.128 (1)	15.987 (3)
*b* (Å)	16.358 (3)	15.202 (3)
*c* (Å)	14.990 (3)	22.151 (4)
β (°)	92.52 (3)	108.36 (3)
Theta range (°)	18.67–1.34	26.02–1.36
R-merge	0.105	0.049
I/s (I)	4.44/1.96	11.6/4.14
Completeness (%)	94.6/96.2	94.3/91.5
Mosaicity (°)	0.24	0.21
Volume (Å^3^)	1501.2	5109.4
Z	2	4
Calculated density (g/cm^3^)	1.53	1.44
Data/restraints/parameters	2274/331/410	9951/0/715
*R*1 indices (I > 2s(I))	0.0999 (0.1582, all data)	0.0591 (0.0881, all data)
*wR2*	0.2468 (0.2921, all data)	0.1631 (0.1867, all data)
*F*(000)	712	2304
Largest diff. peak and hole	0.58 and −0.38 e^−^/Å^3^	1.47 and −0.49 e^−^/Å^3^
Goodness-of-fit on F^2^	1.02	1.03

## Data Availability

Not applicable.

## Supplementary Materials

The following are available online: Figure S1: Job’s plot for the L-zinc (II) adduct (*x* = [Metal]/[Metal] + [Ligand]). Figure S2: Fluorescence spectra of L2Zn in the presence of 1 equivalent of interferent transition metal ions in 200 mM ethanolic solutions. In the inset: quantitative representation by the interferogram curve.
