# Supplementary material for: A Novel L-Shaped Fluorescent Probe for AIE Sensing of Zinc (II) Ion by a DR/NIR Response
Source: Molecules. 2021 Dec 3;26(23):7347. doi: 10.3390/molecules26237347 (PMC8658931; doi:10.3390/molecules26237347)
Supplement: Supplementary file 1 [file molecules-26-07347-s001.zip › molecules-1472739-supplementary.pdf]

A Novel L-Shaped Fluorescent Probe for AIE Sensing of Zinc (II) Ion by a DR/NIR Response

Rosita Diana [1], Ugo Caruso [2], Francesco Silvio Gentile [2], Luigi Di Costanzo [1] and Barbara Panunzi [1,*]

[1] Department of Agriculture, University of Napoli Federico II, 80055 Portici, NA, Italy; rosita.diana@unina.it (R.D.); luigi.dicostanzo4@unina.it (L.D.C.)
[2] Department of Chemical Sciences, University of Napoli Federico II, 80126 Napoli, Italy; ugo.caruso@unina.it (U.C.); francesco.gentile@unina.it (F.S.G.)
* Correspondence: barbara.panunzi@unina.it; Tel.: +39-081-674-170

SUPPLEMENTARY MATERIAL

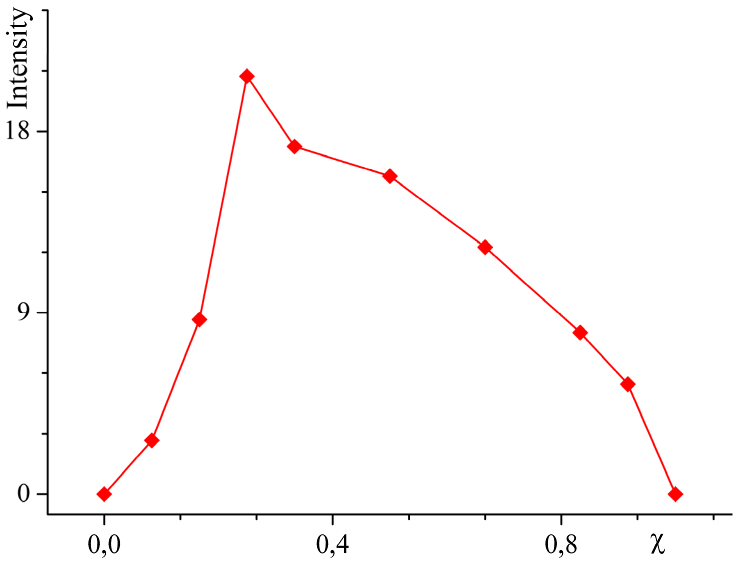

Figure S1. Job's plot for the L-zinc (II) adduct (χ = [Metal]/[Metal]+[Ligand]).

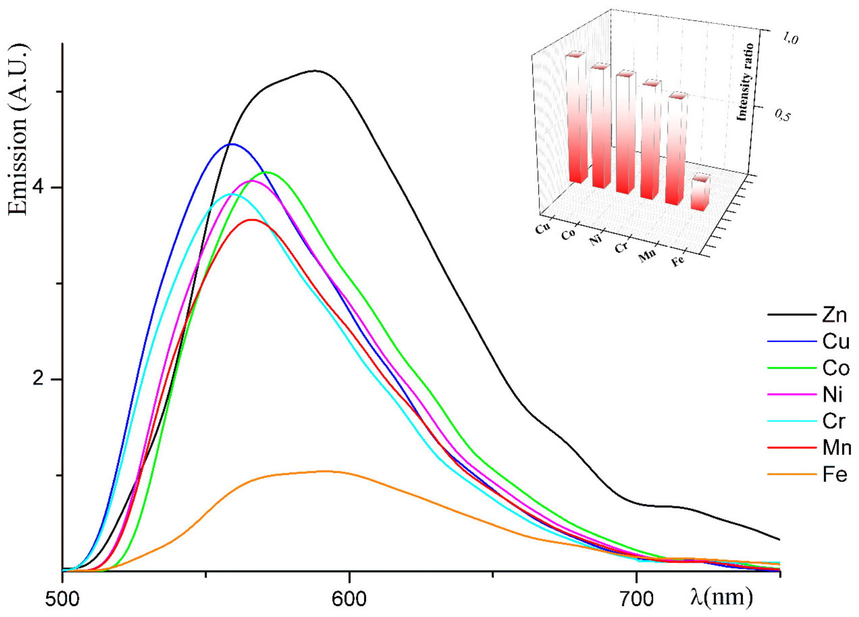

Figure S2. Fluorescence spectra of L_2Zn in presence of 1 equivalent of interferent transition metal ions in 200 mM ethanolic solutions. In the inset: quantitative representation by the interferogram curve.

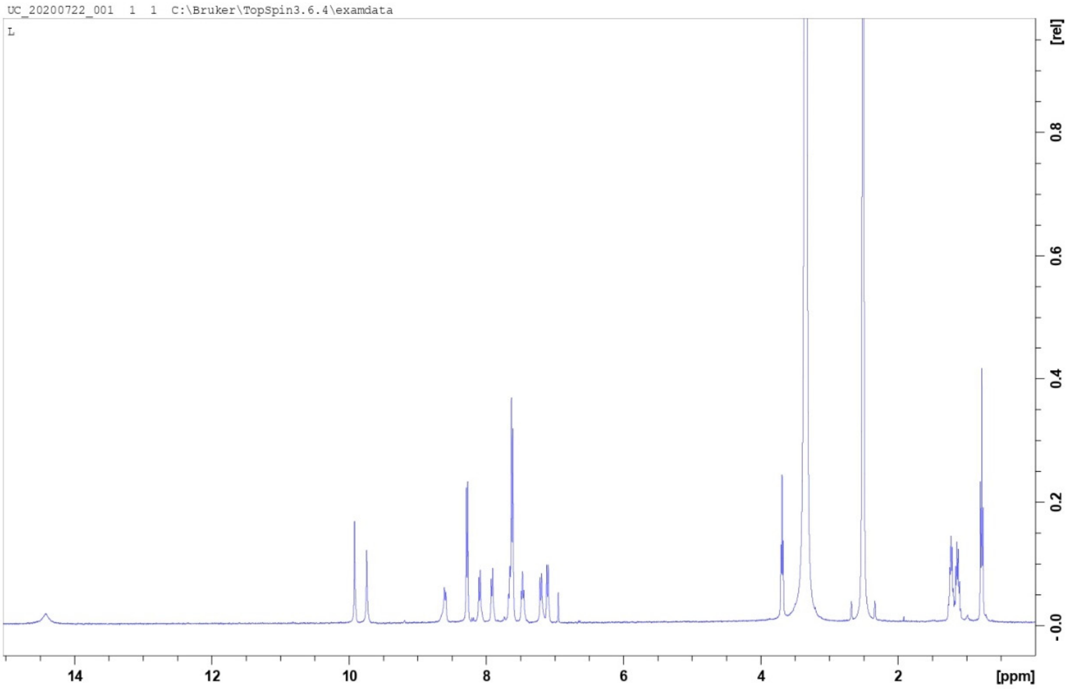

Hard copy of the ^{1}H NMR (400 MHz, DMSO-d_6, 25 ◦C, ppm) spectrum of L.

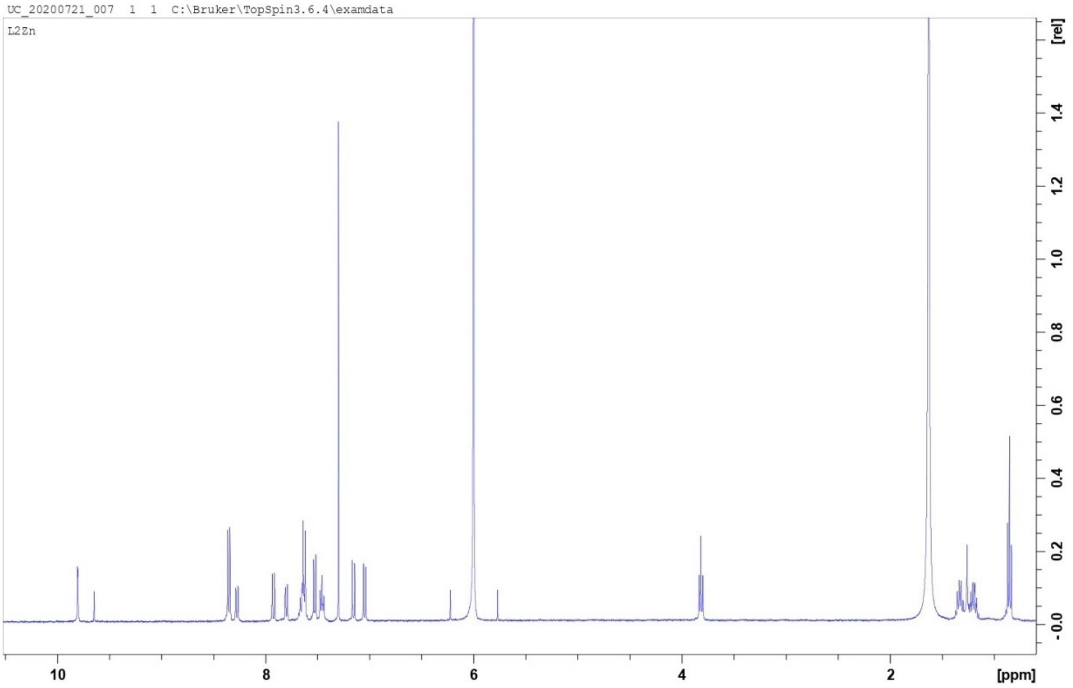

Hard copy of the ^{1}H NMR (400 MHz, DMSO-d_6, 25 ◦C, ppm) spectrum of L_2Zn.
